# Percutaneous transhepatic biliary stenting in patients with intradiverticular papillae and biliary strictures caused by ampullary carcinoma: A case report

**DOI:** 10.3892/ol.2014.1821

**Published:** 2014-01-24

**Authors:** HONG-TAO NIU, QIANG HUANG, REN-YOU ZHAI

**Affiliations:** 1 Department of Interventional Radiology, The First Hospital of Qinhuangdao, Qinhuangdao, Hebei 066000, P.R. China; 2 Department of Interventional Radiology, Beijing Chaoyang Hospital, The Affiliated Hospital of Capital Medical University, Beijing 100020, P.R. China

**Keywords:** intradiverticular papilla, percutaneous transhepatic biliary stenting, malignant biliary stricture, endoscopic retrograde cholangiopancreatography

## Abstract

Endoscopic retrograde cholangiopancreatography with endoscopic sphincterotomy is a well-established procedure for the treatment of bile duct strictures. However, the procedure is difficult to perform in patients with intradiverticular papillae or tumor infiltration of the major papilla. Percutaneous transhepatic biliary stenting (PTBS) is commonly used in the management of malignant biliary stricture. The current study reports two cases of PTBS performed to treat malignant obstructive jaundice caused by ampullary carcinoma complicated with intradiverticular papillae. PTBS is potentially a safe technique for this relatively rare condition.

## Introduction

Endoscopic retrograde cholangiopancreatography (ERCP) is the mainstay treatment for numerous patients with malignant obstructive jaundice. However, in specific cases, it is difficult to perform when the main duodenal papilla infiltrated by the tumor is endoscopically inaccessible. The main duodenal papilla has been found inside a diverticulum in 5–23% of patients in an ERCP study series (1). Therefore, intradiverticular papillae are also one of the causes of the endoscopic inaccessibility in the main duodenal papilla.

Percutaneous transhepatic biliary drainage (PTBD) with stent insertion is an established procedure for the palliation of patients with malignant biliary strictures. The current case report describes percutaneous transhepatic biliary stenting (PTBS) performed in two patients with malignant biliary strictures caused by tumor infiltration of the major papilla and intradiverticular papilla, a rarely reported situation.

## Case report

### Patient 1

The first patient was an 87-year-old male who presented with a one-week history of obstructive jaundice. Abdominal CT showed an ampullary mass surrounding the pancreatic head with a large juxtapapillary diverticulum, as well as the dilation of the extra- and intra-hepatic bile duct. ERCP revealed intradiverticular papilla, bile flow into the diverticulum from the major papilla and a nodular lesion around the diverticulum, with the formation of a shallow ulcer. Cannulation of the major papilla through ERCP failed. However, a biopsy was obtained from the nodule lesion and a well-differentiated adenocarcinoma was confirmed by pathological analysis. The patient was of advanced age and refused surgery. PTBS was performed. The patient recovered well following the procedure and was discharged from the hospital three days after surgery.

### Patient 2

**Figure 1:**
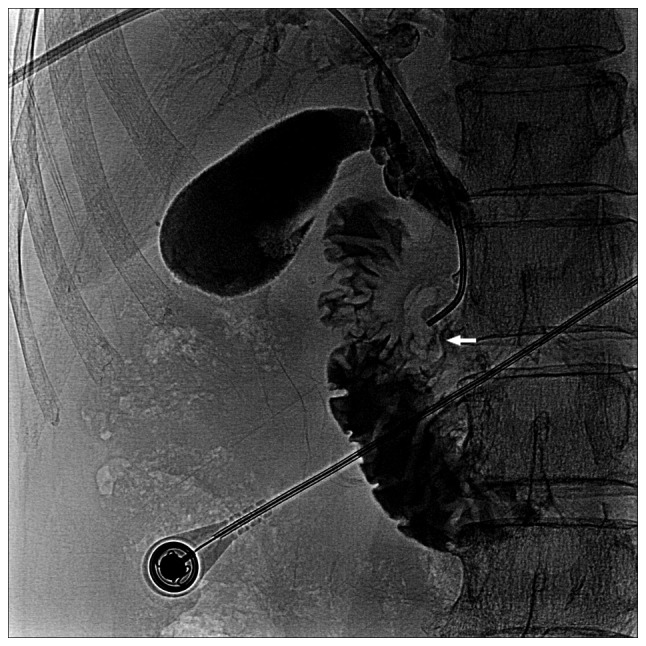
Image showing the injection of contrast agent into the diverticulum through a catheter, confirming mucosal opacification (white arrow) outside the duodenum.

**Figure 2:**
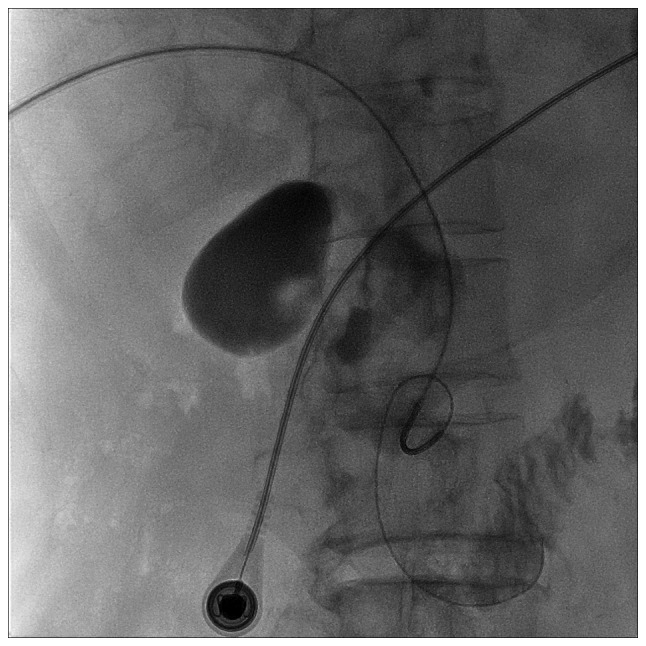
Guidewire being circled in the lumen of the diverticulum and passed through the orifice of the diverticulum into the distal segment of the duodenum during the PTBS procedure. PTBS, percutaneous transhepatic biliary stenting.

**Figure 3:**
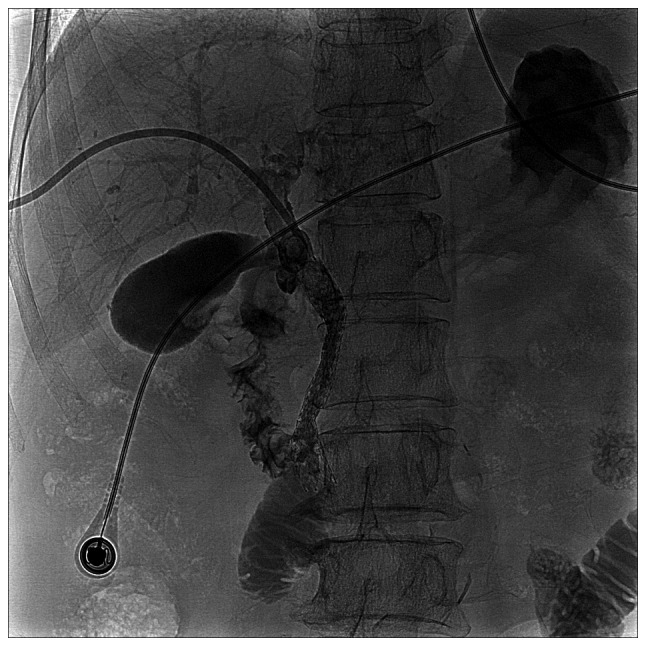
A self-expanding metallic stent was inserted alongside the guidewire and through the papilla into the duodenum. Cholangiography reveals contrast agent passing through the stent into the duodenum.

**Figure 4:**
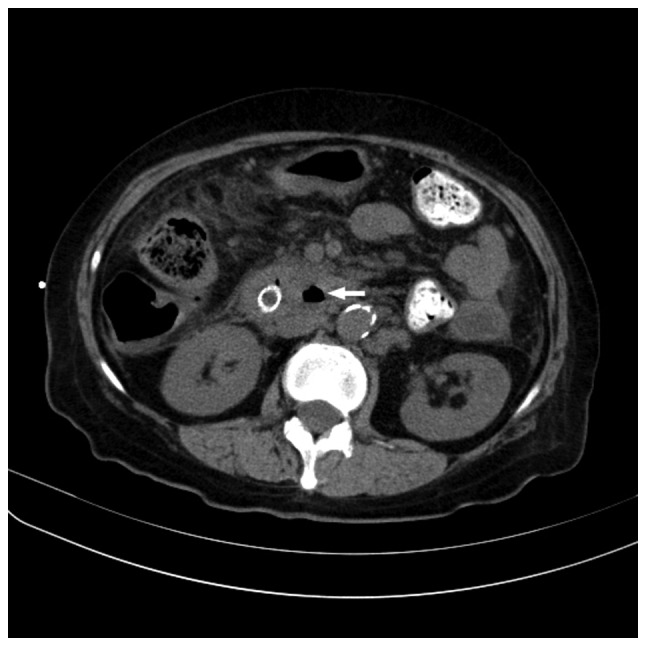
CT performed 4 days after PTBS revealing a tumor surrounding the intradiverticular papilla (white arrow) and the bile duct stent. PTBS, percutaneous transhepatic biliary stenting.

The second patient was a 68-year-old female referred to the Department of Interventional Radiology, Beijing Chaoyang Hospital (Beijing, China) by an endoscopist following failed ERCP. The patient refused surgery. Adenocarcinoma of the ampulla was confirmed by biopsy. PTBS was performed to resolve obstructive jaundice. The major papilla was located inside a large juxtapapillary diverticulum during the procedure (Figs. 1–2). The biliary stent was implanted successfully to cover the stenotic bile duct, with perfect cholangiographic results (Fig. 3). The patient experienced an uneventful post-procedural outcome. CT performed 4 days after PTBS showed the tumor surrounding the intradiverticular papilla (Fig. 4).

### PTBS

Written informed consent was obtained prior to the interventional procedure in the two patients. PTBD was performed using right lobe punctures under fluoroscopic guidance in each patient. Access to the biliary tree was gained using standard interventional techniques and a 7F sheath was inserted to facilitate the following procedure. A 5F, 40-cm angled tip catheter (Kumpe; William Cook Europe ApS, Bjaeverskov, Denmark) was used in conjunction with a 0.035-inch hydrophilic guidewire (Radiofocus M; Terumo Corporation, Tokyo, Japan) to advance through the papillary stricture into the duodenum. The hydrophilic guidewire was exchanged for a 0.035-inch stiff guidewire (Amplatz Super Stiff; Boston Scientific, Natick, MA, USA). A 0.8×4.0-cm balloon (William Cook Eurpoe ApS) was advanced over the guidewire towards the duodenum to dilate the stricture and papilla orifice. Finally, a self-expanding metallic stent (Zilver; William Cook Eurpoe ApS) was inserted alongside the guidewire and through the papilla into the duodenum.

PTBS was performed successfully without procedure-associated complications in the two patients. The patients remained well at the six-month post-procedure follow-up visit.

## Discussion

ERCP with endoscopic sphincterotomy is a well-established procedure for the treatment of bile duct strictures. ERCP with stent insertion in patients with malignant pancreatic-biliary strictures has a success rate between 70 and 95% (2). However, endoscopic stent placement is difficult and in specific cases, is impossible in patients who have intradiverticular papillae or tumor involvement of the papillae (2). Failed therapeutic ERCP due to deep cannulation of the obstructed bile duct is precluded by severe duct angulation, a tight stricture or infiltration by the tumor, and have been previously reported (3,4). ERCP with modified techniques to treat obstructive jaundice in patients with intradiverticular papilla has been described in the literature (2,4–8).

PTBD with stent insertion is a well-established technique for treatment of malignant biliary obstructions (9). Identification and selective cannulation of the papillary orifice via the percutaneous transhepatic route is a relatively simple process even when biliary obstruction due to tumor infiltration occurs. The distortion of the lower common bile duct caused by the duodenal diverticulum enables the duct to form an acute angle as it enters the duodenum. Technical modification and careful manipulation is required to ensure success and avoid complications when malignant obstructive jaundice caused by ampullary carcinoma is combined with intradiverticular papillae. The present case report of two patients describes the interventional procedures to gain access to the bile duct. A 5F angled tip catheter used in conjunction with a soft, angled tip guidewire allows successful cannulation of the intradiverticular papilla, while reducing the risk of diverticular perforation. A stiff guidewire was exchanged to allow the exertion of greater force and to facilitate the insertion of balloon catheter and stent catheter for balloon dilation and stent insertion. Duodenal mucosa opacification inside the diverticulum and duodenum is necessary to confirm the correct location and route of the catheter (Fig. 1).

No case of PTBS has been reported in the literature in patients with intradiverticular papilla and malignant obstructive jaundice caused by ampullary carcinoma. Intradiverticular papillae are not uncommon and must have been identified during previous PTBD or PTBS procedures, however, to the best of our knowledge, there is no prior case report of this event. This may be due to the relative feasibility and safety of the procedure with percutaneous transhepatic methods to pass the intradiverticular papilla. PTBS appears to be forgotten in the literature on treatment of malignant obstructive jaundice with intradiverticular papilla in the era of endoscopy.

An advantage of endoscopic biliary drainage compared with external PTBD is an improved quality of life due to the internal placement of the stent (2). However, the quality of life of patients with PTBS is comparable to that of patients undergoing ERCP, since internal drainage through biliary stent insertion may be performed. Further comparative study of PTBS verses ERCP is required to evaluate the technical and treatment outcomes.

In conclusion, PTBS is potentially a safe and feasible technique for patients with an endoscopically inaccessible intradiverticular papilla and malignant obstructive jaundice caused by ampullary carcinoma. Endoscopists and interventional radiologists must be aware of this under-reported occurrence and offer appropriate management options to patients.

## References

[1] Külling D, Haskell E (2005). Double endoscope method to access intradiverticular papilla. Gastrointest Endosc.

[2] Tarantino I, Barresi L, Fabbri C, Traina M (2012). Endoscopic ultrasound guided biliary drainage. World J Gastrointest Endosc.

[3] Brauer BC, Chen YK, Fukami N, Shah RJ (2009). Single-operator EUS-guided cholangiopancreatography for difficult pancreaticobiliary access (with video). Gastrointest Endosc.

[4] Mallery S, Matlock J, Freeman ML (2004). EUS-guided rendezvous drainage of obstructed biliary and pancreatic ducts: report of 6 cases. Gastrointest Endosc.

[5] Burmester E, Niehaus J, Leineweber T, Huetteroth T (2003). EUS-cholangio-drainage of the bile duct: report of 4 cases. Gastrointest Endosc.

[6] Kahaleh M, Wang P, Shami VM, Tokar J, Yeaton P (2005). EUS-guided transhepatic cholangiography: report of 6 cases. Gastrointest Endosc.

[7] Kim YS, Gupta K, Mallery S, Li R, Kinney T, Freeman ML (2010). Endoscopic ultrasound rendezvous for bile duct access using a transduodenal approach: cumulative experience at a single center. A case series. Endoscopy.

[8] Rajnakova A, Goh PM, Ngoi SS, Lim SG (2003). ERCP in patients with periampullary diverticulum. Hepatogastroenterology.

[9] Garcarek J, Kurcz J, Guzinski M, Janczak D, Sasiadek M (2012). Ten years single center experience in percutaneous transhepatic decompression of biliary tree in patients with malignant obstructive jaundice. Adv Clin Exp Med.

